# VMF3/GPT3: refined discrete and empirical troposphere mapping functions

**DOI:** 10.1007/s00190-017-1066-2

**Published:** 2017-09-15

**Authors:** Daniel Landskron, Johannes Böhm

**Affiliations:** Technische Universität Wien, Vienna, Austria

**Keywords:** VLBI, GNSS, Troposphere, Mapping functions, Horizontal gradients

## Abstract

Incorrect modeling of troposphere delays is one of the major error sources for space geodetic techniques such as Global Navigation Satellite Systems (GNSS) or Very Long Baseline Interferometry (VLBI). Over the years, many approaches have been devised which aim at mapping the delay of radio waves from zenith direction down to the observed elevation angle, so-called mapping functions. This paper contains a new approach intended to refine the currently most important discrete mapping function, the Vienna Mapping Functions 1 (VMF1), which is successively referred to as Vienna Mapping Functions 3 (VMF3). It is designed in such a way as to eliminate shortcomings in the empirical coefficients *b* and *c* and in the tuning for the specific elevation angle of $3^{\circ }$. Ray-traced delays of the ray-tracer RADIATE serve as the basis for the calculation of new mapping function coefficients. Comparisons of modeled slant delays demonstrate the ability of VMF3 to approximate the underlying ray-traced delays more accurately than VMF1 does, in particular at low elevation angles. In other words, when requiring highest precision, VMF3 is to be preferable to VMF1. Aside from revising the discrete form of mapping functions, we also present a new empirical model named Global Pressure and Temperature 3 (GPT3) on a $5^{\circ }\times 5^{\circ }$ as well as a $1^{\circ }\times 1^{\circ }$ global grid, which is generally based on the same data. Its main components are hydrostatic and wet empirical mapping function coefficients derived from special averaging techniques of the respective (discrete) VMF3 data. In addition, GPT3 also contains a set of meteorological quantities which are adopted as they stand from their predecessor, Global Pressure and Temperature 2 wet. Thus, GPT3 represents a very comprehensive troposphere model which can be used for a series of geodetic as well as meteorological and climatological purposes and is fully consistent with VMF3.

## Introduction

During their passage through the neutral atmosphere, radio waves are delayed and bent as a result of interaction with dry gases and water particles. As there is no chance to directly measure these delays with sufficient accuracy, they need to be modeled. The common concept for this purpose is to determine the delay in zenith direction and multiply it with a mapping function intended to scale it to the elevation angle of the observation. Because the composition of atmospheric matter fluctuates heavily both temporally and spatially, values for these zenith delays and mapping functions are ever changing. One of the most accurate ways of obtaining (at least approximate values of) troposphere delays is ray-tracing through numerical weather models (NWMs). In these numerical weather models, the lower atmosphere is discretized to a specific horizontal grid resolution and a number of height levels through which the ray-tracing beams then propagate. They are delayed and bent following the complex laws of refraction what is thought to approximate the real travel path as well as possible. For this reason, ray-tracing is, unlike surface measurement methods, able to consider the effect of the whole atmosphere. Current ray-tracing software such as RADIATE (Hofmeister and Böhm 2017) manages to compute ray-traced delays for a more or less limited number of observations such as those from VLBI ($\sim$10 million since advent in 1979); however, it is evidently not possible in terms of computational effort to do this for every single GNSS observation. The concept of mapping functions provides remedy as the information of the variability of delays over the whole elevation range is condensed in them, more precisely in three mapping function coefficients *a*, *b* and *c*. The first mapping function to adopt information from ray-tracing through NWMs was the Isobaric Mapping Functions (IMF) by Niell (2000), which induced a major leap in accuracy at that time. Böhm and Schuh (2004) drew on this concept and devised the Vienna Mapping Functions (VMF) which overcame some limitations of IMF, especially in the wet part. The subsequent Vienna Mapping Functions 1 (VMF1) by Böhm et al. (2006a) is regarded as the most accurate mapping functions nowadays and are applied by numerous research centers and other agencies worldwide. While VMF1 is retrieving data from the European Centre for Medium-Range Weather Forecasts (ECMWF), the UNB-VMF1 (Santos et al. 2012) does the same for NWM data from the United States National Centers for Environmental Prediction (NCEP) and the Canadian Meteorological Center (CMC). Also other models such as the Adaptive Mapping Functions (AMF) by Gegout et al. (2011) or the Potsdam Mapping Factors (PMF) by Zus et al. (2014) are based on the concept of ray-tracing through NWMs.

Mapping functions adopting information from ray-tracing through NWMs at certain times and locations are commonly referred to as discrete mapping functions in this paper. In contrast, empirical troposphere models and mapping functions rely on experience values from climatology and are of vital importance particularly for all applications that do not have internet connection and thus have no possibility of downloading the latest discrete data. Also applications which simply do not require utmost accuracy benefit from empirical troposphere delay models owing to their straightforward usage. Their accuracy is certainly lower than that of discrete mapping functions which harness real observation data, but yet they are frequently used in all space geodetic techniques. Important realizations of empirical troposphere models and mapping functions are (in chronological order) the New Mapping Functions (NMF) by Niell (1996), the Global Mapping Functions (GMF) by Böhm et al. (2006b), the model UNB3m (Leandro et al. 2006) or the models Global Pressure and Temperature 2 (GPT2) by Lagler et al. (2013) and its successor Global Pressure and Temperature 2 wet (GPT2w) by Böhm et al. (2015), having improved capability to determine zenith wet delays empirically. However, the modeling of troposphere delays still leaves considerable room for improvement, which is why there remains large interest in ever more accurate troposphere delay modeling techniques both for discrete and for empirical purposes.

## Fundamentals of troposphere modeling

Following Nilsson et al. (2013), the total delay time $\Delta L(\varepsilon )$ which radio waves experience when traveling through the neutral atmosphere depending on the observation elevation angle $\varepsilon$ is commonly modeled with the parametrization in Eq. (1) (Davis et al. 1985):

1 $$\begin{aligned} \Delta L(\varepsilon ) = \Delta L_\mathrm{h}^\mathrm{z} \cdot \hbox {mf}_\mathrm{h}(\varepsilon ) + \Delta L_\mathrm{w}^\mathrm{z} \cdot \hbox {mf}_\mathrm{w}(\varepsilon ) \end{aligned}$$

The delay modeling is obviously split into a hydrostatic part, which is mainly caused by the dry gases in the atmosphere, and a wet part which arises from water vapor and water particles in the atmosphere, each represented through a multiplication of the respective delay in zenith direction $\Delta L^\mathrm{z}$ with a mapping function $\hbox {mf}(\varepsilon )$. The zenith hydrostatic delay $\Delta L_\mathrm{h}^\mathrm{z}$ can be determined with very high precision through pressure measurements at the site, as the weight of all air layers adds up to the surface pressure. The equation by Saastamoinen (1972) as revised by Davis et al. (1985)

2 $$\begin{aligned} \Delta L_\mathrm{h}^\mathrm{z} = \frac{0.0022768 \cdot p}{1-0.00266 \cdot \cos (2\varphi )-0.28 \cdot 10^{-6} \cdot h_\mathrm{ell}} \end{aligned}$$

is generally used for this purpose, where *p* is the pressure and $\varphi$ and $h_\mathrm{ell}$ the geographic latitude and ellipsoidal height of the station, respectively. Deriving the zenith wet delay $\Delta L_\mathrm{w}^\mathrm{z}$ is far more difficult because surface measurements alone are not sufficient for this. Common practice in space geodesy is to estimate this parameter in the analysis on the basis of a sufficient overdetermination of observations, which, however, is not always given. An approach to approximate $\Delta L_\mathrm{w}^\mathrm{z}$ is the formula by Askne and Nordius (1987) which requires three input parameters: water vapor pressure *e*, mean temperature weighted with water vapor pressure $T_\mathrm{m}$ and water vapor decrease factor $\lambda$:

3 $$\begin{aligned} \Delta L_\mathrm{w}^\mathrm{z} = 10^{-6} \cdot \bigg ( k_2' + \frac{k_3}{T_\mathrm{m}} \bigg ) \cdot \frac{R_\mathrm{d} \cdot e}{g_\mathrm{m} \cdot (\lambda +1)} \end{aligned}$$

$k_2'$ and $k_3$ represent empirically determined refractivity constants here, while $R_\mathrm{d}$ is the specific gas constant for dry constituents which equals 287.0464 $\hbox {JK}^{-1}\,\hbox {kg}^{-1}$ and $g_\mathrm{m}$ is the mean gravity which equals 9.80665 $\hbox {ms}^{-2}$. Apart from that, ray-tracing through numerical weather models is also capable of computing very precise values for both $\Delta L_\mathrm{h}^\mathrm{z}$ and $\Delta L_\mathrm{w}^\mathrm{z}$ (Teke et al. 2011).

Following the model by Marini (1972) in its truncated form by Herring (1992), which is the basis for all “modern” mapping functions developed so far, the $\hbox {mf}(\varepsilon )$ are each built up on the basis of three coefficients *a*, *b* and *c*:

4 $$\begin{aligned} \hbox {mf}(\varepsilon ) = \frac{1+\frac{a}{1+\frac{b}{1+c}}}{\sin (\varepsilon )+\frac{a}{\sin (\varepsilon )+\frac{b}{\sin (\varepsilon )+c}}} \end{aligned}$$

According to Herring (1992), *a*, *b* and *c* are defined as coefficients that depend on integrals of refractivity through the atmosphere. To put it another way, mapping functions can also be regarded as a measure of the thickness of the neutral atmosphere. With decreasing thickness compared to the earth’s radius (as is the case at the poles for instance), the coefficients decrease and the mapping function approaches $\sin (\varepsilon )^{-1}$ (Niell 2000). On account of its principal order in the formula, the coefficient *a* is the determining element of Eq. (4). In all discrete mapping function approaches mentioned in Introduction, the information from the NWMs is incorporated into the coefficient *a*, while *b* and *c* rely on empirical functions. In empirical mapping functions also the *a* coefficients are of empirical nature.

The general purpose of this paper is to conceptualize mapping functions which are able to surpass the performance of VMF1 and GPT2w, which are considered among the most accurate mapping functions of their kind. For this purpose, in the following an understanding of the general concept of these two is given.

The Vienna Mapping Functions 1 (VMF1) is a model providing discrete values for zenith hydrostatic delay $\Delta L_\mathrm{h}^\mathrm{z}$, zenith wet delay $\Delta L_\mathrm{w}^\mathrm{z}$ and the hydrostatic and wet mapping functions $\hbox {mf}_\mathrm{h}$ and $\hbox {mf}_\mathrm{w}$. Therein the coefficients $b_\mathrm{h}$, $b_\mathrm{w}$ and $c_\mathrm{w}$ are constants, while $c_\mathrm{h}$ is dependent on day of year (doy) and geographic latitude. The hydrostatic and wet *a* coefficients are determined directly from ray-traced delays at the initial elevation angle $3.3^{\circ }$ through inverting Eq. (4). This is done for each NWM epoch, that is, daily at 00:00, 06:00, 12:00 and 18:00 UT for a specific set of stations as well as on a global grid. The respective values at the observation epoch can eventually be obtained through interpolation from adjacent NWM epochs. In addition, Böhm et al. (2009) developed the VMF1-FC which provides the VMF1 coefficients also up to two days in advance and thus opened the possibility of using VMF1 for real-time applications.

Global Pressure and Temperature 2 wet (GPT2w) is an empirical model for troposphere delays which is the successor of the former models GPT (Böhm et al. 2007) and GPT2 (Lagler et al. 2013). It requires only information about time and location and provides mean values plus annual and semi-annual amplitudes of a set of quantities such as mapping function coefficients $a_\mathrm{h}$ and $a_\mathrm{w}$, temperature *T*, pressure *p*, water vapor pressure *e*, mean temperature weighted with water vapor pressure $T_\mathrm{m}$ and water vapor decrease factor $\lambda$, optionally on a $5^{\circ }\times 5^{\circ }$ and a $1^{\circ }\times 1^{\circ }$ grid. The coefficients were derived from monthly mean pressure-level data of ERA-Interim fields by the ECMWF.

**Table 1:** A list of all mapping function approaches mentioned throughout this paper

Identifier	name
$\hbox {VMF1}_{\mathrm{original}}$	Vienna Mapping Functions 1
$\hbox {VMF1}_{\mathrm{repro3deg}}$	Reprocessed VMF1; empirical *b* and *c* (Böhm et al. 2006a), *a* for $3^{\circ }$ (outgoing) elevation
$\hbox {VMF1}_{\mathrm{reproLSM}}$	Reprocessed VMF1; empirical *b* and *c* (Böhm et al. 2006a), *a* from LSM
$\hbox {VMF}_{\mathrm{LSM}}$	*a*, *b* and *c* from LSM
$\hbox {VMF3}_{\mathrm{3deg}}$	Vienna Mapping Functions 3; empirical *b* and *c* (this paper), *a* for $3^{\circ }$ (outgoing) elevation
$\hbox {VMF3}_{\mathrm{LSM}}$	Vienna Mapping Functions 3; empirical *b* and *c* (this paper), *a* from LSM
GPT2w	Global Pressure and Temperature 2 wet (optionally on a $5^{\circ }\times 5^{\circ }$ or $1^{\circ }\times 1^{\circ }$ grid)
GPT3	Global Pressure and Temperature 3 (optionally on a $5^{\circ }\times 5^{\circ }$ or $1^{\circ }\times 1^{\circ }$ grid)

For all LSM versions, 7 (outgoing) elevation angles are used ($3^{\circ }$, $5^{\circ }$, $7^{\circ }$, $10^{\circ }$, $15^{\circ }$, $30^{\circ }$, $70^{\circ }$)

## Development of new mapping functions

As the publication of VMF1 dates back to 2006, many new approaches have evolved over the years, however none of which was actually able to outperform VMF1 yet. Nonetheless, Zus et al. (2015) revealed shortcomings in VMF1 due to its tuning for the specific elevation angle of $3^{\circ }$, station heights and orbital altitudes. For those reasons, it was tried to conceive a new, however similar mapping function concept to overcome these problems. NWMs have improved significantly since 2006 what made it possible to draw on a much larger data framework for this purpose. The new discrete mapping function is to be named VMF3, following the draft VMF2 (Böhm et al. 2005) which has never become operational as it was not able to sufficiently improve the results of VMF1. Analogously, the data are also used for designing a new empirical mapping function consecutively named Global Pressure and Temperature 3 (GPT3) that is also assumed to benefit from the higher amount of data and is fully consistent with VMF3.

In the course of this paper, a series of new models with separate names is designed and tested, which might create confusion as they all resemble each other. Therefore, Table 1 lists all names and labels to serve as a guide. The theory behind each approach is to be explained in the upcoming sections; however, it is stated already at this point that the final products of this section will be the methods $\hbox {VMF3}_{\mathrm{LSM}}$ and GPT3.

### Vienna Mapping Functions 3

What is striking in Table 1 is that there are least-squares method (LSM) approaches and non-LSM approaches. The idea behind this is that with the ray-tracer RADIATE programmed in FORTRAN it is possible to create millions of ray-traced delays in virtually no time at all, so the mapping function coefficients can be determined efficiently from ray-tracing at not just a single elevation angle, such as in VMF1, but also from a number of elevation angles through least-squares methods. The reprocessed VMF1 coefficients ($\hbox {VMF1}_{\mathrm{repro3deg}}$) are calculated based on exactly the same model, but new ray-tracing data. This shall allow estimations about the quality of the ray-tracing data itself on the one hand, and comparisons which would not be possible with the $\hbox {VMF1}_{\mathrm{original}}$ on the other hand. The following subsections are intended to explain the theory behind each approach.

#### $\hbox {VMF1}_{\mathrm{repro3deg}}$ and $\hbox {VMF1}_{\mathrm{reproLSM}}$

Here the *b* and *c* coefficients are adopted from VMF1, while the *a* coefficients get new values based on the ray-tracing data whose properties are listed in Table 3.

For $\hbox {VMF1}_{\mathrm{repro3deg}}$, RADIATE is used to compute the mapping function $\hbox {mf}(3^\circ )$ for each observation which is then, together with the empirical *b* and *c*, inserted into the following formula in order to analytically calculate *a*:

5 $$\begin{aligned} a = -\frac{\hbox {mf}(\varepsilon ) \cdot \sin (\varepsilon ) - 1}{\frac{\hbox {mf}(\varepsilon )}{\sin (\varepsilon ) + \frac{b}{\sin (\varepsilon )+c}} - \frac{1}{1+\frac{b}{1+c}}} \end{aligned}$$

This is done separately for the hydrostatic and the wet part. For $\hbox {VMF1}_{\mathrm{reproLSM}}$, the situation is different because the *a* coefficients are fitted to ray-traced mapping function coefficients at the whole elevation range, which requires least-squares adjustments. Because the equation system is nonlinear, in fact (unweighted) iterative least-squares adjustments must be applied employing starting values of $a_{\mathrm{h}_0} = 0.0012$, $a_{\mathrm{w}_0} = 0.00055$, although the adjustment is very insensitive to the choice of the starting values; even using the (absolutely unrealistic) starting values $a_{\mathrm{h}_0} = 0.005$, $a_{\mathrm{w}_0} = 0.002$ instead does not change the results at all. Convergence is assumed as soon as the additions are smaller than $10^{-12}$ which corresponds to an accuracy of the resulting delay of approximately $6 \times 10^{-9}$ m. For details, see Landskron (2017).

#### $\hbox {VMF}_{\mathrm{LSM}}$

Here all three mapping function coefficients are determined together in least-squares adjustments. This appears to be the best approach of simulating the ray-traced delays, because the coefficients then contain the full information of the NWMs and do not suffer from sometimes better, sometimes worse fitting empirical parameters. The iterative adjustment requires starting values also for *b* and *c*, which are set to $b_{\mathrm{h}_0} = 0.0029$, $b_{\mathrm{w}_0} = 0.00146$, $c_{\mathrm{h}_0} = 0.065$ and $c_{\mathrm{w}_0} = 0.04391$. At first glance, it seems as if this would be the best mapping function concept; however, for two reasons it cannot be used operationally:

- Convergence of the wet coefficients can only be achieved when the underlying NWM is sufficiently “smooth”. This means that the ray-traced delays must exhibit a more or less linear variation over the elevation angles, otherwise the iterative LSM immediately diverges. For the hydrostatic part, this is no problem at all, but the wet delays are affected by too many small-scale variations so that it is not possible to determine $a_\mathrm{w}$, $b_\mathrm{w}$ and $c_\mathrm{w}$ for discrete locations and times from operational NWM data. Small-scale variations in the wet delay at different elevation angles certainly represent important information about the actual state of the troposphere; however, they conflict with the determination of single coefficients which shall represent the state at all elevation angles. For a global grid based on monthly averaged NWM values, the situation is different as the upcoming Sect. 3.1.3 addresses.
- According to Böhm (2004), the interpolation, which has to be performed separately for each of the three coefficients *a*, *b* and *c* by the user, involves danger because of the inherent high correlation between them.

#### $\hbox {VMF3}_{\mathrm{3deg}}$ and $\hbox {VMF3}_{\mathrm{LSM}}$

The only way to improve the VMF1 concept when *b* and *c* have to keep on their empirical nature is to significantly improve and extend the underlying empirical model. The coefficients $b_\mathrm{h}$, $b_\mathrm{w}$, $c_\mathrm{h}$ and $c_\mathrm{w}$ all need to be equipped with spatial as well as temporal variation components on whose basis $a_\mathrm{h}$ and $a_\mathrm{w}$ can be computed.

As mentioned before, the determination of $\hbox {VMF}_{\mathrm{LSM}}$ does not fail for monthly mean NWMs in which all meteorological quantities are strongly smoothed. For the operational provision of mapping functions, this obviously does not make sense; however, it enables the determination of discrete *b* and *c* values on a grid from which, in a further step, empirical information can be derived. Hence, ray-traced delays are produced on a global $5^{\circ }\times 5^{\circ }$ grid, monthly for the time period of 2001 to 2010 (Table 2) from which the $\hbox {VMF}_{\mathrm{LSM}}$ coefficients are then estimated.

**Table 2:** Properties of the grid-wise ray-traced delays that were generated for the derivation of VMF3

Parameter	Specification
Ray-tracing software	RADIATE (Hofmeister and Böhm 2017)
Ray-tracing method	2D piecewise linear (Hobiger et al. 2008)
NWM	ECMWF ERA-Interim Pressure-Level Data
Horizontal resolution of the NWM	$1^{\circ }\times 1^{\circ }$
Horizontal coverage	(1) global grid with resolution $5^{\circ }\times 5^{\circ }$ (lat: [$87.5^{\circ }$, $-87.5^{\circ }$], lon: [$2.5^{\circ }$, $357.5^{\circ }$]), resulting in 2592 grid points and (2) global grid with resolution $1^{\circ }\times 1^{\circ }$ (lat: [$89.5^{\circ }$, $-89.5^{\circ }$], lon: [$0.5^{\circ }$, $359.5^{\circ }$]) resulting in 64800 grid points
Vertical coverage	25 Pressure levels
Temporal resolution	Mean values for every month from 2001 through 2010 ($=$ 120 epochs)
Outgoing elevation angles per point	4 ($3.3^{\circ }$, $5^{\circ }$, $15^{\circ }$ and $30^{\circ }$) for $5^{\circ }\times 5^{\circ }$ grid and 1 elevation ($3^{\circ }$) for $1^{\circ }\times 1^{\circ }$ grid
Azimuth angles per point	8 ($0^{\circ }$:$45^{\circ }$:$315^{\circ }$)

**Fig. 1:**
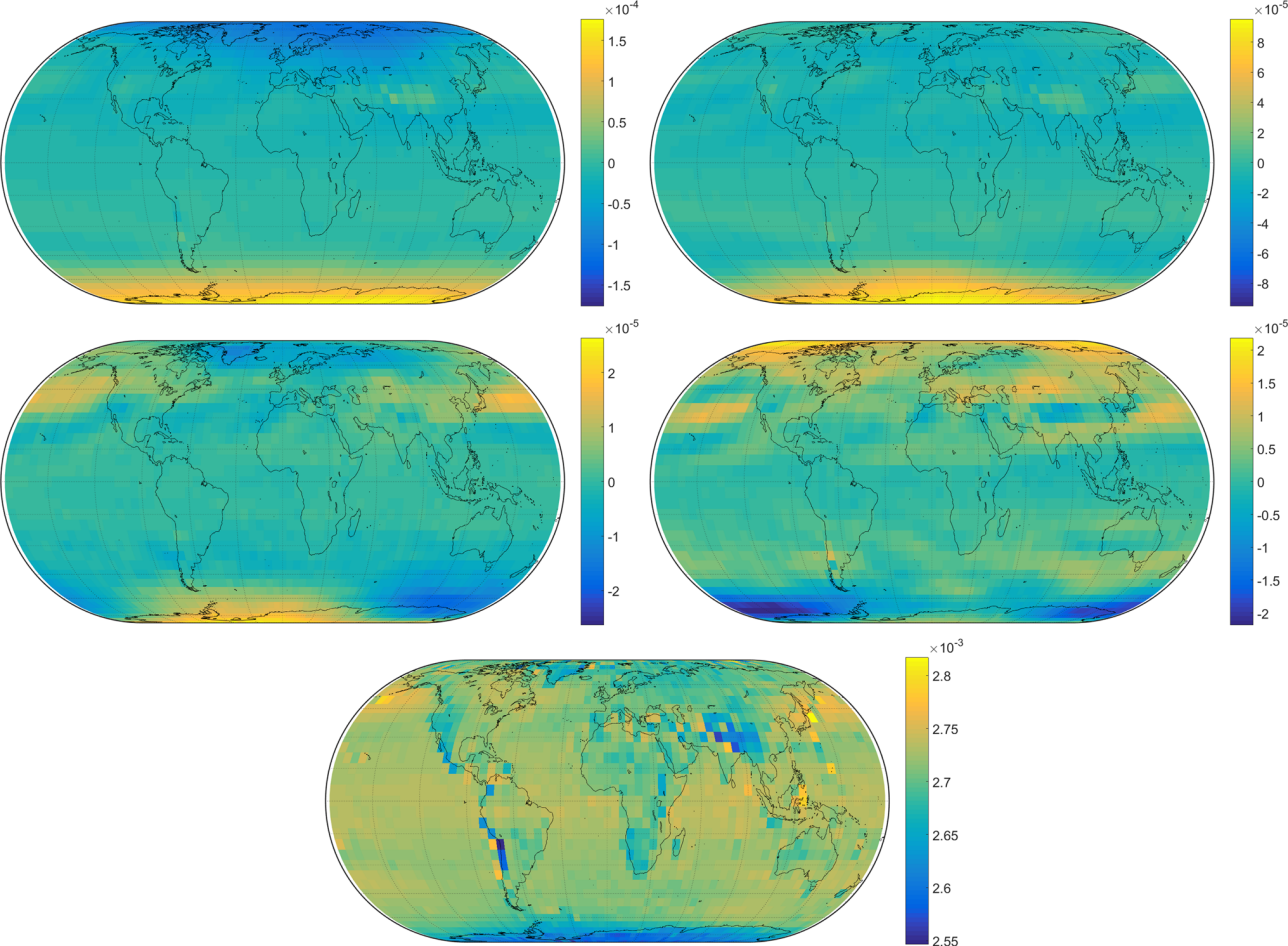
Parameters of the seasonal fit for the mapping function coefficient $b_\mathrm{h}$. Top left: annual amplitude $A_1$, top right: annual amplitude $B_1$, center left: semi-annual amplitude $A_2$, center right: semi-annual amplitude $B_2$ and bottom: mean values $A_0$

In order to deduce empirical temporal information for the coefficients *b* and *c*, the following seasonal fit formula is applied (Lagler et al. 2013; Böhm et al. 2015). For $b_\mathrm{h}$, it would appear as:

6 $$\begin{aligned} \begin{aligned} b_\mathrm{h}&= A_0 + A_1\cdot \cos \left( \frac{\hbox {doy}}{365.25}2\pi \right) + B_1\cdot \sin \left( \frac{\hbox {doy}}{365.25}2\pi \right) \\&\quad +\, A_2\cdot \cos \left( \frac{\hbox {doy}}{365.25}4\pi \right) + B_2\cdot \sin \left( \frac{\hbox {doy}}{365.25}4\pi \right) \end{aligned} \end{aligned}$$

in which $A_0$ represents the mean value, $A_1$ and $B_1$ the annual amplitudes and $A_2$ and $B_2$ the semi-annual amplitudes of the coefficient. Least-squares adjustments are again used to fit these parameters to the $\hbox {VMF}_{\mathrm{LSM}}$ data. Figure 1 contains the results for the coefficient $b_\mathrm{h}$.

The coefficients and their amplitudes could be saved as a grid, from which the user then could spatially interpolate the desired position. However, this would be accompanied with unacceptably long loading times, particularly for a range of positions and times. Therefore, it was decided to represent the discrete grid by continuous functions, which is accomplished through spherical harmonics, which are commonly used for representations of the geoid and the gravitational and magnetic fields of the Earth. In fact, $b_\mathrm{h}$, $b_\mathrm{w}$, $c_\mathrm{h}$ and $c_\mathrm{w}$ and their amplitudes must pass through another least-squares adjustment in order to be fitted to the spherical harmonics coefficients. For details of the spherical harmonics estimation, it is again referenced to Landskron (2017).

**Fig. 2:**
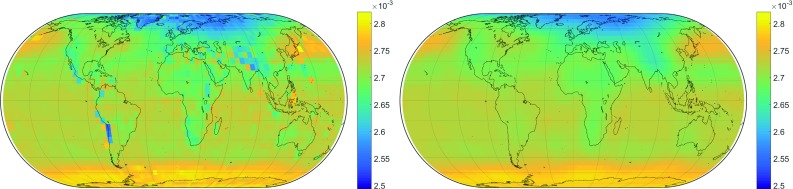
Empirical coefficient $b_\mathrm{h}$ at the arbitrary epoch January 15, 2001 (MJD: 51924). Left: the original grid which is to be represented by spherical harmonics. Right: spherical harmonics representation for degree of expansion $n=12$

Setting the degree of expansion to $n = m = 12$, 91 Legendre coefficients must be estimated by LSM for each mapping function coefficient and each of its amplitudes. Figure 2 shows the results of the spherical harmonics expansion exemplarily for a certain time, compared to the original grid. In general, the representation works very well. For small-scale variations such as over mountain ranges like the Himalayas or the Andes, the degree of expansion $n=12$ is obviously too low, which, however, is not critical because small errors in the *b* and *c* coefficients can be compensated by the *a* coefficients.

The empirical coefficients $b_\mathrm{h}$, $b_\mathrm{w}$, $c_\mathrm{h}$ and $c_\mathrm{w}$ now have appropriate temporal and spatial variations that are considerably more advanced than those of VMF1. Using these, discrete values for $a_\mathrm{h}$ and $a_\mathrm{w}$ can be determined. As mentioned already at an earlier stage, this is handled—once more—through a least-squares adjustment over all seven elevation angles for the representation $\hbox {VMF3}_{\mathrm{LSM}}$ and for the single outgoing elevation angle of $3^{\circ }$ for version $\hbox {VMF3}_{\mathrm{3deg}}$. The performance of all approaches introduced throughout this chapter ($\hbox {VMF1}_{\mathrm{repro3deg}}$, $\hbox {VMF1}_{\mathrm{reproLSM}}$, $\hbox {VMF3}_{\mathrm{3deg}}$ and $\hbox {VMF3}_{\mathrm{LSM}}$) is assessed in Sect. 4.

### Global Pressure and Temperature 3

**Fig. 3:**
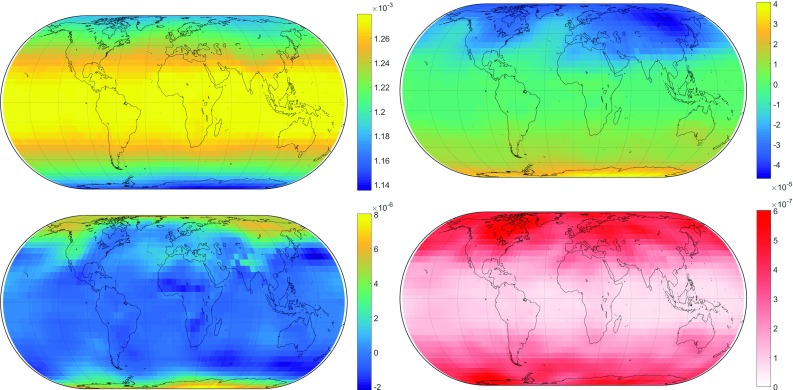
Mean values $A_0$ (top left), seasonal amplitudes $A_1$ (top right), half-seasonal amplitudes $A_2$ (bottom left) and standard deviation of the residuals of $A_0$ (bottom right) of the hydrostatic mapping function coefficient $a_\mathrm{h}$ from GPT3. At a rough estimate, given the uncertainty of $6 \times 10^{-7}$ in $A_0$ and of $8 \times 10^{-7}$ in all amplitudes of $a_\mathrm{h}$ (as is the case at the poles), the resulting slant hydrostatic delay at $5^{\circ }$ elevation would change at worst by 4 mm

In the previous section, empirical representations of the mapping function coefficients $b_\mathrm{h}$, $b_\mathrm{w}$, $c_\mathrm{h}$ and $c_\mathrm{w}$ were found from which the discrete $a_\mathrm{h}$ and $a_\mathrm{w}$ can be calculated. To create an all-empirical mapping function model, these need to be represented empirically as well. Having done most of the groundwork already through the generation of VMF3, the only step remaining is to apply Eq. (6) to the discrete *a* coefficients. The resulting values are then stored in a grid, as is done in GPT2w, while *b* and *c* retain their spherical harmonics expression. The crucial difference is that for the empirical version the $a_\mathrm{h}$ coefficients must be valid at sea level instead of the respective height of the topography so that users can then reproduce them for any location on earth, because the magnitude of $a_\mathrm{h}$ is dependent on ellipsoidal height $h_\mathrm{ell}$. The height correction by Niell (1996) is the suitable tool for handling this:

7 $$\begin{aligned} \begin{aligned} \hbox {mf}_{\mathrm{h}_0} =&\,\hbox {mf}_{\mathrm{h}_1} - \frac{h_\mathrm{ell}}{1000} \\&\times \left( \frac{1}{\sin (\varepsilon )} - \frac{ 1+\frac{a_{\mathrm{h}t}}{1+\frac{b_{\mathrm{h}t}}{1+c_{\mathrm{h}t}}} }{ \sin (\varepsilon )+\frac{a_{\mathrm{h}t}}{\sin (\varepsilon )+\frac{b_{\mathrm{h}t}}{\sin (\varepsilon )+c_{\mathrm{h}t}}} } \right) \end{aligned} \end{aligned}$$

where $\hbox {mf}_{\mathrm{h}_0}$ is the hydrostatic mapping function at reduced height 0 (usually sea level), $\hbox {mf}_{\mathrm{h}_1}$ is the hydrostatic mapping function at height 1 (usually at the topography), and the constants $a_{\mathrm{h}t} = 2.53 \times 10^{-5}$, $b_{\mathrm{h}t} = 5.49 \times 10^{-3}$ and $c_{\mathrm{h}t} = 1.14 \times 10^{-3}$ define the correction. Figure 3 depicts the resulting $a_\mathrm{h}$ coefficients on the grid.

**Table 3:** Properties of the station-wise ray-traced delays that were generated using the ray-tracer RADIATE from 1999 to 2014

Parameter	Specification
Ray-tracing software	RADIATE (Hofmeister and Böhm 2017)
Ray-tracing method	2D piecewise linear (Hobiger et al. 2008)
NWM	ECMWF ERA-Interim Pressure-Level Data + ECMWF operational data
Horizontal resolution of the NWM	$1^{\circ }\times 1^{\circ }$
Vertical coverage	25 Pressure levels
Horizontal coverage	33 VLBI stations
Temporal resolution	6-hourly at 00:00, 06:00, 12:00 and 18:00 UTC each day from 1999 through 2014 ($=$23376 epochs)
Outgoing elevation angles per point	7 ($3^{\circ }$, $5^{\circ }$, $7^{\circ }$, $10^{\circ }$, $15^{\circ }$, $30^{\circ }$ and $70^{\circ }$)
Azimuth angles per point	16 ($0^{\circ }$:$22.5^{\circ }$:$337.5^{\circ }$)

**Table 4:** Mean absolute error (first column), mean bias (second column) and mean standard deviation (third column) in slant total delay $\Delta L$ at $5^{\circ }$ elevation (mm) between ray-tracing and several mapping function approaches, averaged over all 2592 grid points and 120 epochs

Trop. model	MAE $\Delta L$	Bias $\Delta L$	$\sigma \Delta L$
$\hbox {VMF}_{\mathrm{LSM}}$	0.35	0.00	0.43
$\hbox {VMF1}_{\mathrm{repro3deg}}$	1.73	0.58	1.23
$\hbox {VMF1}_{\mathrm{reproLSM}}$	1.49	0.50	1.08
$\hbox {VMF3}_{\mathrm{3deg}}$	0.93	$-$0.04	0.84
$\hbox {VMF3}_{\mathrm{LSM}}$	0.82	$-$0.03	0.73
GMF	10.21	$-$2.08	10.47
GPT2w	6.85	0.32	8.26
GPT3	6.44	$-$1.03	7.98

The several meteorological quantities from GPT2w are left unchanged for GPT3. They are of particular importance for creating empirical zenith delays; pressure *p* can be converted to zenith hydrostatic delay $\Delta L_\mathrm{h}^\mathrm{z}$ using Eq. (2), while inserting water vapor pressure *e*, mean temperature weighted with water vapor pressure $T_\mathrm{m}$, and water vapor decrease factor $\lambda$ into Eq. (3) produces empirical zenith wet delay $\Delta L_\mathrm{w}^\mathrm{z}$. In addition, the ray-traced delays are also utilized for determining an empirical gradient grid capable of outperforming currently existing models. Thus, a full empirical troposphere model is provided. The empirical gradient grid, however, is not part of this paper; for more information see Landskron (2017). The eventual GPT3 troposphere model is realized on a $5^{\circ }\times 5^{\circ }$ as well as on a $1^{\circ }\times 1^{\circ }$ grid, which is naturally assumed to be more precise, and consists of the quantities listed in Table 8.

**Fig. 4:**
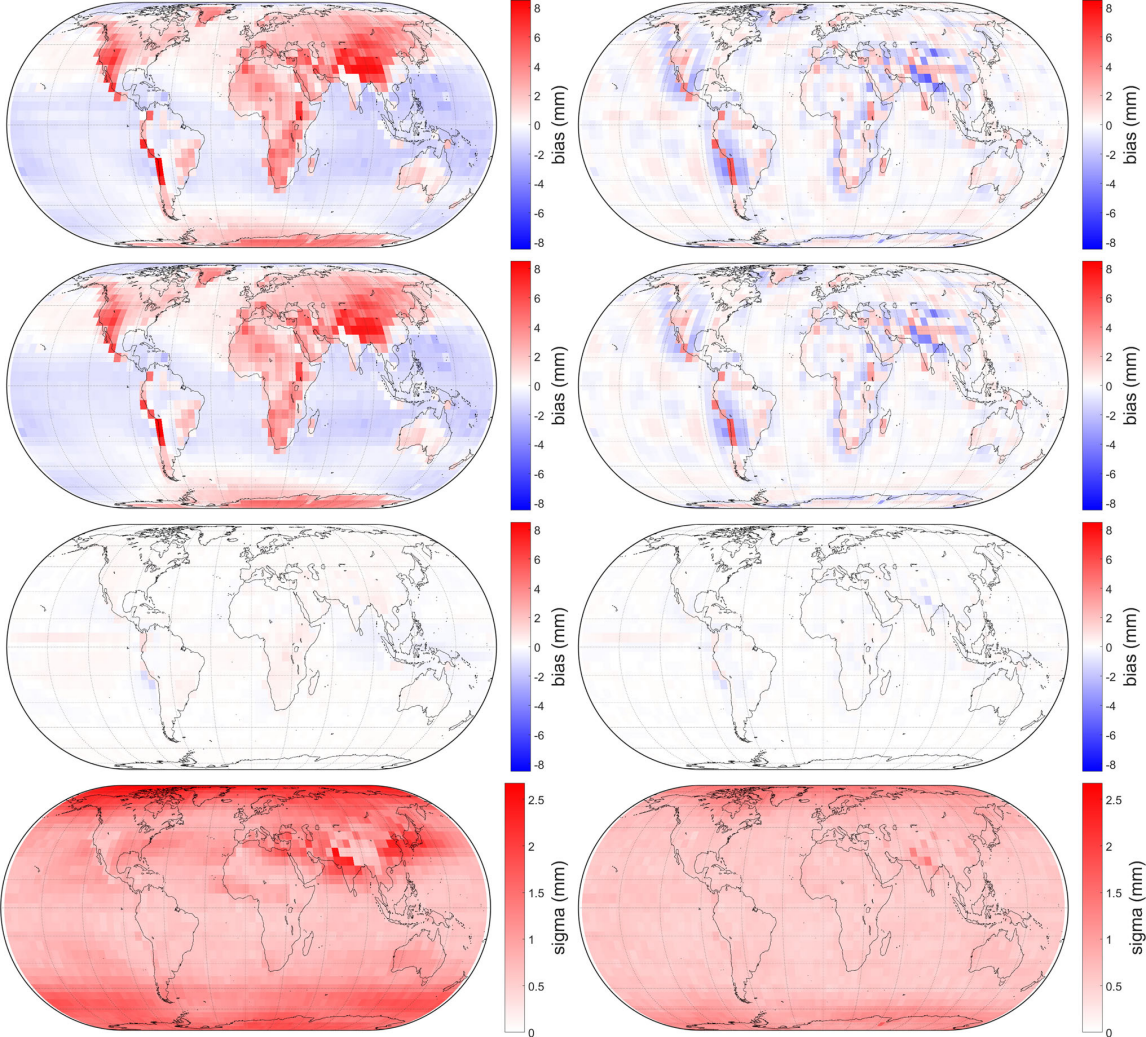
Differences in slant delays at $5^{\circ }$ elevation between $\hbox {VMF1}_{\mathrm{repro3deg}}$ (left) and $\hbox {VMF3}_{\mathrm{LSM}}$ (right) to the ray-traced delays, averaged over all 120 epochs. Top: bias in slant total delay $\Delta L$, center top: bias in slant hydrostatic delay $\Delta L_\mathrm{h}$, center bottom: bias in slant wet delay $\Delta L_\mathrm{w}$ and bottom: standard deviation in slant total delay $\Delta L$

**Fig. 5:**
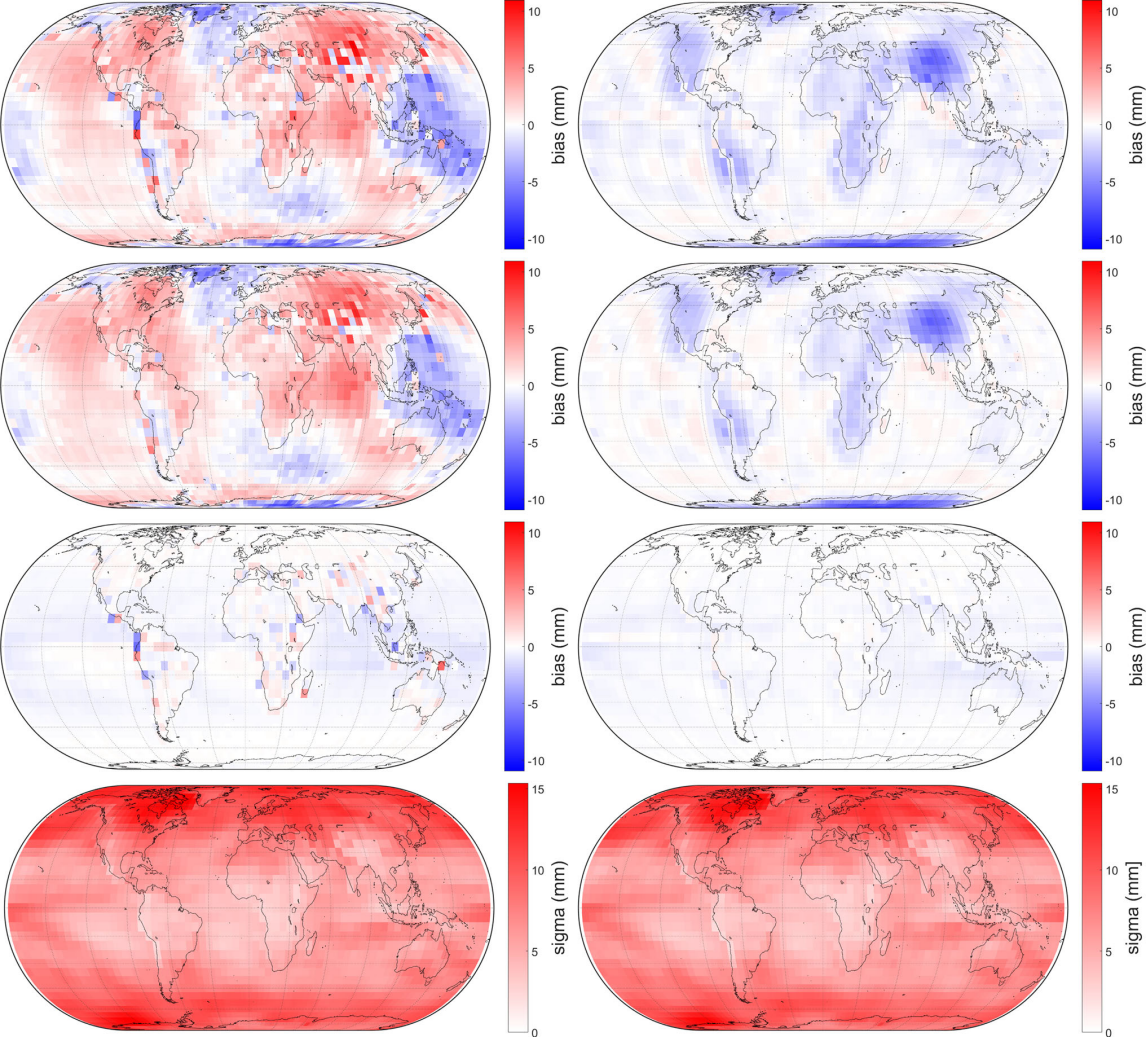
Differences in slant delays at $5^{\circ }$ elevation between GPT2w (left) and GPT3 (right) to the ray-traced delays, averaged over all 120 epochs. Top: bias in slant total delay $\Delta L$, center top: bias in slant hydrostatic delay $\Delta L_\mathrm{h}$, center bottom: bias in slant wet delay $\Delta L_\mathrm{w}$ and bottom: standard deviation in slant total delay $\Delta L$

**Table 5:** Mean absolute error (first column), mean bias (second column) and mean standard deviation (third column) in slant total delay $\Delta L$ at $5^{\circ }$ elevation (mm) between ray-tracing and several mapping function approaches, averaged over all 33 stations and epochs from 1999 to 2014

Trop. model	MAE $\Delta L$	Bias $\Delta L$	$\sigma \Delta L$
$\hbox {VMF1}_{\mathrm{original}}$	8.30	0.72	12.71
$\hbox {VMF1}_{\mathrm{repro3deg}}$	3.98	2.66	4.24
$\hbox {VMF1}_{\mathrm{reproLSM}}$	3.47	2.32	3.71
$\hbox {VMF3}_{\mathrm{3deg}}$	2.97	1.72	3.57
$\hbox {VMF3}_{\mathrm{LSM}}$	2.64	1.58	3.15
GPT2w ($5^{\circ }\times 5^{\circ }$)	18.95	-0.53	24.74
GPT2w ($1^{\circ }\times 1^{\circ }$)	18.90	-0.21	24.69
GPT3 ($5^{\circ }\times 5^{\circ }$)	18.98	-2.43	24.69
GPT3 ($1^{\circ }\times 1^{\circ }$)	18.84	0.20	24.53

**Table 6:** Mean absolute error (first column), mean bias (second column) and mean standard deviation (third column) in slant total delay $\Delta L$ at $3^{\circ }$ elevation (mm) between ray-tracing and several mapping function approaches, averaged over all 33 stations and epochs from 1999 to 2014

Trop. model	MAE $\Delta L$	Bias $\Delta L$	$\sigma \Delta L$
$\hbox {VMF1}_{\mathrm{original}}$	22.19	$-$5.42	33.88
$\hbox {VMF1}_{\mathrm{repro3deg}}$	0.52	0.00	0.64
$\hbox {VMF1}_{\mathrm{reproLSM}}$	1.62	$-$1.02	1.75
$\hbox {VMF3}_{\mathrm{3deg}}$	0.52	0.00	0.64
$\hbox {VMF3}_{\mathrm{LSM}}$	1.17	$-$0.45	1.50
GPT2w ($5^{\circ }\times 5^{\circ }$)	54.35	$-$5.34	70.09
GPT2w ($1^{\circ }\times 1^{\circ }$)	54.13	$-$4.38	69.93
GPT3 ($5^{\circ }\times 5^{\circ }$)	54.49	$-$8.22	70.21
GPT3 ($1^{\circ }\times 1^{\circ }$)	53.86	$-$0.41	69.58

## Results

In the following, two comparisons are described to assess the performance of VMF3 and GPT3 relative to other approaches: (1) baseline length repeatabilities (BLRs) from VLBI analyses using the Vienna VLBI Software (VieVS) (Böhm et al. 2012) are compared, and (2) the modeled delays are compared to those of ray-tracing, which are regarded as the true delays for this purpose. For the BLR comparison, only station-wise data (Table 3) are employed, while for the delay comparison both station-wise and grid-wise data (cf. Table 2) are regarded, but separately.

The BLR is the standard deviation of a set of baseline lengths between two stations. These stations are also subject to plate motions and other discontinuities over the long term, which must be corrected beforehand so that only the error of the modeling approach remains. The lower the standard deviation, the better the modeling. However, it turned out that the different mapping functions produce only marginal differences in baseline lengths, with empirical mapping functions even yielding results equivalent to the discrete ones. Thus, comparing BLRs is not sufficient for assessing differences between mapping functions [for details see Landskron (2017)].

**Fig. 6:**
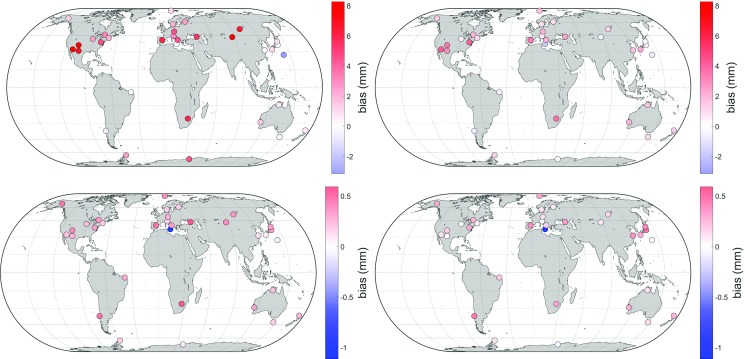
Mean differences in slant hydrostatic delays (top) and slant wet delays (bottom) at $5^{\circ }$ elevation between $\hbox {VMF1}_{\mathrm{repro3deg}}$ (left) and $\hbox {VMF3}_{\mathrm{LSM}}$ (right) to the ray-traced delays. $\hbox {VMF3}_{\mathrm{LSM}}$ outperforms $\hbox {VMF1}_{\mathrm{repro3deg}}$ at 27 of the 33 stations in hydrostatic delay and at all stations, albeit only marginally, in wet delay

A more effective comparison among the mapping functions is provided by comparing the delays directly. The better the modeled delays approximate the ray-traced delays, the higher their quality is, when considering the ray-traced delays as the true reference values. For all tested approaches, the zenith delays from RADIATE are used so that differences can be attributed solely to the mapping factors.

First, the comparison is done for the global grid which was already used for the creation of VMF3 and GPT3. Table 4 shows the results on the basis of comparisons of mean absolute error (MAE), mean bias and mean standard deviation. $\hbox {VMF1}_{\mathrm{original}}$ cannot be included here as it is not available for the chosen grid.

From this, it can be concluded that $\hbox {VMF}_{\mathrm{LSM}}$, the approach where all three coefficients *a*, *b* and *c* are estimated in the least-squares adjustment, gets closest to the ray-traced delays. However, for the reasons mentioned in Sect. 3.1.2, this approach is not suitable for station-wise application. The delays from $\hbox {VMF3}_{\mathrm{3deg}}$ are not far off of those from $\hbox {VMF}_{\mathrm{LSM}}$, but considerably better than those from $\hbox {VMF1}_{\mathrm{repro3deg}}$. The VMF3 approach obviously outperforms the VMF1 approach, while the estimation through LSM yields a further small improvement. Figure 4 illustrates this graphically.

VMF3 reduces apparent shortcomings of the VMF1 approach in particular in mountainous areas, which almost exclusively appear in the hydrostatic part. Also, as evident from Table 4 and shown in Fig. 5, delays modeled with GPT3 are closer to the ray-traced delays than those modeled with GPT2w. The improvement of GPT3 over GPT2w is, in fact, not as distinct as it appears to be in the figure because the delay differences were averaged over all 120 epochs before and thus lost their positive or negative algebraic signs. Besides, on the global grid it makes no difference whether the $5^{\circ }\times 5^{\circ }$ or $1^{\circ }\times 1^{\circ }$ versions of GPT2w and GPT3 are used, since either of them exactly coincides exactly with the global grid points.

The second comparison of delay differences is made for 15 years of data (cf. Table 3) for 33 VLBI stations all around the globe which were chosen in such a way as to reach a global distribution that is as uniform as possible. Tables 5 and 6 show the resulting differences between the modeled delays and the reference ray-traced delays for the two elevation angles $5^{\circ }$ and $3^{\circ }$. The zenith delays again come from RADIATE for all model approaches so that differences in the slant delays merely stem from differences in mapping factors. Figure 6 shows the improvement of $\hbox {VMF3}_{\mathrm{LSM}}$ over $\hbox {VMF1}_{\mathrm{repro3deg}}$ at each station. $\hbox {VMF1}_{\mathrm{original}}$ is also stated in this comparison; as it is determined from entirely different ray-traced delays, however, the values are not necessarily representative.

Also from these tables and figures, it is obvious that the VMF3 approach outperforms the VMF1 approach. At $3^{\circ }$ elevation, the non-LSM version is best, but this is no surprise since the *a* coefficients were determined for this very elevation angle. At $5^{\circ }$ elevation (and all other larger elevation angles, which are not included in the tables), however, the LSM version is superior. Consequently, the approach $\hbox {VMF3}_{\mathrm{LSM}}$ is regarded as the best result. In all comparisons, the bulk of improvement comes from the hydrostatic part, while the wet part does not differ significantly. The empirical model GPT3 is apparently able to marginally exceed GPT2w in the $1^{\circ }\times 1^{\circ }$ version, but not in the $5^{\circ }\times 5^{\circ }$ version. For geodetic purposes, the effect of the mapping function on station positions is most important. A rule of thumb says that the error in the height component is approximately one-fifth of the delay error at an elevation angle of $5^{\circ }$ (Böhm 2004); this means that station heights are improved by 0.25 mm when using $\hbox {VMF3}_{\mathrm{LSM}}$ instead of $\hbox {VMF1}_{\mathrm{repro3deg}}$. Concerning empirical mapping functions, there is virtually no station height change.

## Conclusions

In this paper, two new mapping function models for troposphere modeling are introduced, one for discrete purposes and one for empirical purposes. The former is referred to as VMF3 (corresponding to the approach $\hbox {VMF3}_{\mathrm{LSM}}$ in the text) and is characterized by a new, more sophisticated handling of the empirical coefficients *b* and *c* compared to VMF1, as well as *a* coefficients which were determined through least-squares adjustments over seven elevation angles. In particular, at low elevation angles VMF3 is able to approximate the underlying ray-traced delays appreciably better than VMF1. At $5^{\circ }$ elevation, the delays are improved on average by 1.3 mm, which is equivalent to an improved station height of 0.25 mm. At higher elevation angles, though, there is not much of a difference between VMF1 and VMF3. For this reason, it depends on the task whether the use of VMF3 is justified or not; for high-precision applications, it certainly makes sense; however for others VMF1 may be sufficient. The ability of empirical models to approximate ray-traced delays is obviously somewhat worse. The newly presented model GPT3 uses the same *b* and *c* coefficients as VMF3 and, in case of the $5^{\circ }\times 5^{\circ }$ version, is based on the same ray-tracing data as VMF3. GPT3 ($5^{\circ }\times 5^{\circ }$) achieves equal results to GPT2w ($5^{\circ }\times 5^{\circ }$), while results from GPT3 ($1^{\circ }\times 1^{\circ }$) are slightly better than those of its counterpart, however being a little more time-consuming. However, the main benefit of GPT3 is its full consistency with VMF3. In future, a new height correction for mapping functions will be determined replacing that of Niell (1996), which is expected to further improve GPT3 and its ability to model troposphere delays at positions other than at or close to the surface of the earth.

**Table 7:** A list of all input and output parameters of the discrete mapping function VMF3

Symbol	Name	Unit
Input parameters		
$a_\mathrm{h}$	Hydrostatic mapping function coefficient	–
$a_\mathrm{w}$	Wet mapping function coefficient	–
$\mathrm{mjd}$	Modified Julian date	–
$\varphi$	Geographic latitude	rad
$\lambda$	Geographic longitude	rad
*zd*	Zenith distance ($\pi$-elevation)	rad
Output parameters		
$\hbox {mf}_\mathrm{h}$	Hydrostatic mapping factor	–
$\hbox {mf}_\mathrm{w}$	Wet mapping factor	–

**Table 8:** A list of all input and output parameters of the empirical troposphere model GPT3

Symbol	Name	Unit
Input parameters		
$\mathrm{mjd}$	Modified Julian date	–
$\varphi$	Geographic latitude	rad
$\lambda$	Geographic longitude	rad
$h_\mathrm{ell}$	Ellipsoidal height	m
Output parameters		
*p*	Pressure	hPa
*T*	Temperature	$^{\circ }$C
*dT*	Temperature lapse rate	K km$^{-1}$
$T_\mathrm{m}$	Mean temperature weighted with water vapor pressure	K
*e*	Water vapor pressure	hPa
$a_\mathrm{h}$	Hydrostatic mapping function coefficient (valid at sea level)	–
$a_\mathrm{w}$	Wet mapping function coefficient	–
$\lambda$	Water vapor decrease factor	–
*N*	Geoid undulation	m
$G_{\mathrm{n_h}}$	Hydrostatic north gradient	m
$G_{\mathrm{e_h}}$	Hydrostatic east gradient	m
$G_{\mathrm{n_w}}$	Wet north gradient	m
$G_{\mathrm{e_w}}$	Wet east gradient	m

Unless otherwise stated, all output quantities are valid for the ellipsoidal height $h_\mathrm{ell}$ specified in the input

## Data and code availability

Required MATLAB scripts and data text files containing the respective mapping function coefficients can be downloaded from http://ggosatm.hg.tuwien.ac.at/DELAY/. Information on the usage of the files is found in http://ggosatm.hg.tuwien.ac.at/DELAY/readme.txt. All input and output parameters of VMF3 and GPT3 are summarized in Tables 7 and 8.
